# PReS-FINAL-2184: A randomized trial in new onset juvenile dermatomyositis: prednisone versus prednisone plus cyclosporine versus prednisone plus methotrexate

**DOI:** 10.1186/1546-0096-11-S2-O19

**Published:** 2013-12-05

**Authors:** N Ruperto, A Pistorio, S Knupp Feitosa de Oliveira, R Cuttica, A Ravelli, M Fischbach, B Magnusson, T Avcin, K Brochard, F Corona, G Couillault, F Dressler, V Gerloni, G Sterba, F Zulian, MT Apaz, A Cespedes-Cruz, R Cimaz, C Bracaglia, R Joos, P Quartier, R Russo, M Tardieu, N Wulffraat, S Angioloni, A Martini

**Affiliations:** 1 Istituto G. Gaslini, Genoa, Italy; 2 PRINTO, Genoa, Italy

## Introduction

Data regarding the safety and efficacy of treatment regimens for juvenile dermatomyositis (JDM) tends to be from anecdotal, small, uncontrolled, non-randomized case series.

## Objectives

To find out the treatment regimen associated with the lowest occurrence of flare and the lowest drug related toxicity in juvenile dermatomyositis (JDM).

## Methods

Children with newly diagnosed JDM were randomized in an open fashion to receive: prednisone (PDN) versus PDN plus methotrexate (MTX) versus PDN plus Cyclosporine A (CASA). Primary outcome measures after 6 months of treatment: response rate according to the Paediatric Rheumatology International Trials Organisation (PRINTO) provisional definition of improvement. Primary outcome measures after 24 months of treatment: a) time to inactive disease; b) time to major therapeutic changes because of inefficacy/flare/adverse events; time to flare.

## Results

139 randomized patients were included in the efficacy dataset. There were 82 females (59%) with a median age at onset of 7.4 years (1st-3rd quartiles **4.4-10.6**) and a median disease duration of 2.8 months (1.3-5.3). Frequency of response at 6 months was for 24/47 (51%) for PDN, 32/46 (70%) for PDN+CSA and 32/46 (70%) for PDN+MTX (p 0.032).

There was a statistically significant difference for inactive disease/clinical remission between group 3 (PDN+MTX) versus group 2 (PDN +CSA) and 1 (PDN) combined (Log-Rank test p 0.021 and p 0.012, respectively) and in the time to major therapeutic change (defined as the addition of CSA or MTX or any other disease-modifying anti-rheumatic drug) between group 1 (PDN) versus group 2 (PDN+CSA) and 3 (PDN+MTX) combined (p = 0.009). No statistical significant differences in time to flare (Log-rank test; p = 0.39). The safety analysis showed that group 2 (PDN+CSA) had greater number of adverse events (AE) when compared to group 1 (PDN) and 3 (PDN+MTX) (p = 0.005). Similarly there was a statistically significant increase in the frequency of AE in group 2 (PDN+CSA) when compared to Group 1 (PDN) and 3 (PDN+MTX) in several systems (skin and subcutaneous tissues, gastrointestinal, and general disorders. Infections/infestations) and others (hypertrichosis, hirsutism/hair growth, and abdominal pain). There were no statistically significant differences in serious AE among the 3 groups (p 0.17). There were no deaths.

## Conclusion

Combined therapy with PDN and either CSA or MTX was more effective than with PDN alone. However the safety profile favors the combination with MTX with respect to CSA.

## Disclosure of interest

None declared.

